# Epigenetic Aging and Links Between Childhood Adversity and Cognition in Later Life

**DOI:** 10.1093/geroni/igaf122.2262

**Published:** 2025-12-31

**Authors:** Marrium Mansoor, Benjamin Katz

**Affiliations:** Virginia Tech, Blacksburg, Virginia, United States; Virginia Tech, Blacksburg, Virginia, United States

## Abstract

Adverse childhood events (ACEs) have been linked to worse outcomes throughout life, from poorer academic and workplace performance to compromised physical health, depression and cognitive impairment. Epigenetic age acceleration (EAA) refers to a combined set of factors causing DNA methylation that may help explain these links While these links have been explored in younger and middle aged adults they remain largely unexamined in large, nationally representative samples of older adults. This study utilized data from 3490 older adults in the United States (Mage = 69.5 years, 58.5% female, 75% white) from the Health and Retirement Study. Childhood adversity was measured through an aggregate score including whether participants had trouble with police, repeated a year of school, experienced substance abuse or physical abuse from parents. EAA was quantified using GrimAge, an epigenetic clock that measures the level of DNA methylation to provide an estimate of mortality and morbidity risk. Mediation models were run via PROCESS to test if GrimAge acted as a mediator between ACEs and cognitive performance measured by Serial 7s and Number Series (executive function measures) along with Immediate and Delayed Recall (memory measures). GrimAge fully mediated the association between ACEs and performance on Serial 7s, Number Series, and Delayed Recall, while controlling for age, gender, race, years of education, and depressive symptoms. This supports the hypothesis that childhood adversity may impact cognitive performance in later life through EAA. These results underscore the importance of interventions and policies to support individuals with ACEs as they age.

